# Porcine sapelovirus 2A protein induces mitochondrial-dependent apoptosis

**DOI:** 10.3389/fimmu.2022.1050354

**Published:** 2022-11-25

**Authors:** Chunxiao Mou, Yuxi Wang, Shuonan Pan, Kaichuang Shi, Zhenhai Chen

**Affiliations:** ^1^ College of Veterinary Medicine, Yangzhou University, Yangzhou, Jiangsu, China; ^2^ Joint International Research Laboratory of Agriculture and Agri-Product Safety, the Ministry of Education of China, Yangzhou University, Yangzhou, Jiangsu, China; ^3^ Jiangsu Co-Innovation Center for Prevention and Control of Important Animal Infectious Diseases and Zoonoses, Yangzhou University, Yangzhou, Jiangsu, China; ^4^ Guangxi Center for Animal Disease Control and Prevention, Nanning, Guangxi, China

**Keywords:** *porcine sapelovirus*, apoptosis, mitochondrial pathway, 2A protein, crucial amino acids

## Abstract

Porcine sapelovirus (PSV) is an emerging pathogen associated with symptoms of enteritis, pneumonia, polioencephalomyelitis and reproductive disorders in swine, resulting in significant economic losses. Although PSV is reported to trigger cell apoptosis, its specific molecular mechanism is unclear. In this research, the cell apoptosis induced by PSV infection and its underlying mechanisms were investigated. The morphologic features of apoptosis include nuclear condensation and fragmentation, were observed after PSV infection. The cell apoptosis was confirmed by analyzing the apoptotic rates, caspase activation, and PARP1 cleavage. Caspase inhibitors inhibited the PSV-induced intrinsic apoptosis pathway and reduced viral replication. Among the proteins encoded by PSV, 2A is an important factor in inducing the mitochondrial apoptotic pathway. The conserved residues H48, D91, and C164 related to protease activity in PSV 2A were crucial for 2A-induced apoptosis. In conclusion, our results provide insights into how PSV induces host cell apoptosis.

## Introduction

Porcine sapelovirus (PSV) is a member of the genus *Sapelovirus* in the family *Picornaviridae*. PSV can cause a series symptoms in swine, including acute diarrhea, respiratory distress, skin lesions, encephalitis, and reproductive tract disorders (1). PSV could colonize in the villous epithelial cells of the small intestine, transmitted *via* the fecal-oral route and had a high prevalence in the faeces samples of diarrhoeal pigs (2, 3). PSV can be found in the samples co-infected with porcine parvovirus, classical swine fever virus, porcine reproductive and respiratory disorder syndrome virus, porcine enterovirus, or other viruses (4), which poses a considerable threat to the pig industry. The PSV genome consists of a positive-sense, single-stranded, non-enveloped RNA of 7.5–8.3 k nucleotides (nt). A typical picornavirus genome encodes a polyprotein containing a leader protein (L), four structural proteins (VP1, VP2, VP3, and VP4), and seven nonstructural proteins (2A, 2B, 2C, 3A, 3B, 3C, and 3D) (5). The first strain of PSV (formerly called PEV-8) was isolated more than 50 years ago in the United Kingdom and was considered a member of the historic group of porcine ‘enteroviruses’. Since then, PSV infection has been reported in many other countries, including China, South Korea, Spain, and Brazil (6).

Apoptosis is a specific form of cell death, and it has been shown to be modulated by extrinsic or intrinsic pathways (7). The morphologic and biochemical features of apoptotic cells, including cell shrinkage, nuclear condensation, DNA fragmentation, cell membrane blebbing, and appearance of phosphatidylserine (PS) at the cell surface (8). The cysteine-dependent aspartate-directed proteinases termed caspases that initiate and execute the intracellular specific proteolytic cleavage events, resulting in cleaving proteins and nucleic acid (9, 10). Caspase-9 and caspase-8 are respectively participated in the intrinsic (also known as mitochondrial) and extrinsic (death receptor) apoptotic pathway. They can activate the downstream caspase-3/7 proteins to initiate apoptosis (7). Apoptosis process is regulated by anti- and pro-apoptosis cellular factors. In the intrinsic apoptotic pathway, Bcl-2 and Bcl-xL are anti-apoptotic proteins which contribute to the cell survival, pro-apoptotic factors Bax and Bak are essential for the execution of the mitochondrial apoptosis pathway. Apoptotic stimulation promotes Bax redistribution from the cytoplasm into the mitochondrial membrane, where it promotes the release of cytochrome c (cyto C) to the cytoplasm. These apoptosis-related proteins finally activates the caspase cascade and initiates apoptosis (11).

In general, apoptosis plays an important role in clearing virus-infected cells and maintaining intracellular homeostasis, and it also represents crucial antiviral defense strategies of the host cells (12, 13). However, viruses have developed diverse mechanisms to regulate apoptosis and ensure their own propagation, and many viral proteins actually trigger apoptosis for virus release (14). Recent reports indicated that the 2A protein of picornaviruses induced severe morphological changes in the overexpressed cells, which was similar to those observed in virus-infected cells (15). The 2A proteases of Poliovirus (PV) can induce apoptosis to promote viral replication (16). Meanwhile, Enterovirus 71 (EV71) 2A protein also triggers the eukaryotic translation initiation factor 4G I cleavage and cell apoptosis for virus release (12). Although PSV has been reported to cause apoptosis, the specific molecular mechanisms are unclear. Therefore, our study aimed to investigate the underlying molecular mechanisms of PSV-induced cell apoptosis, thereby providing the theoretical basis for preventing the PSV infection.

## Materials and methods

### Cells, viruses, reagents, and antibodies

ST cells and HEK293A cells were purchased from China Institute for Veterinary Drug and cultured in Dulbecco’s Minimal Essential Medium (DMEM; Hyclone) with 10% fetal bovine serum (FBS; Sigma). All cells were maintained in 37°C 5% CO_2_ incubator. The PSV was isolated from PSV-infected piglets in Hunan Province, China, and kept in our laboratory. Z-VAD-FMK, ZIETD-FMK, and Z-LEHD-FMK obtained from Beyotime. The PSV VP1 protein-specific monoclonal antibody was prepared by our laboratory. The GAPDH, Bax, Bcl-2, and Cyt c antibodies were purchased from ABclonal. Antibodies against caspase-3 (D3R6Y), caspase-8 (1C12) and caspase-9 (Human Specific) were obtained from Cell Signaling Technologies. The antibody against PARP1 was purchased from Beyotime. The anti-Tomm 20 antibody were purchased from Abcam. The antibody against Flag was purchased from Sigma. HRP-goat anti-mouse lgG, HRP-goat anti-rabbit lgG, Alexa Fluor 549-goat anti-mouse lgG, and Alexa Fluor 488-goat anti-mouse lgG antibodies were obtained from Proteintech.

### Construction of plasmids

All fragments corresponding to PSV-encoded proteins were amplified from the PSV cDNA and inserted into pCAGGS-3×Flag. The mutation fragments of 2A gene were obtained by homologous recombination and also inserted into the pCAGGS-3×Flag. These recombinant plasmids were identified by DNA sequencing. Primers designed based on the genomic sequence of PSV (**Table 1**).

**Table 1:** The primers used for construction of plasmids expressing the PSV-encoded proteins and 2A mutants.

Primer	Sequence
PSV-L F	CGCGAATTCAATGGAATCTACTACTACTCTTTCATTTTGC
PSV-L R	GACTGGTACCTTGGGGTTTGTTACCTGTATTAGATGAATG
PSV-VP4 F	CGCGAATTCAGGAGCCTACAACCATGGTTCAG
PSV-VP4 R	GACTGGTACCCTTGAACGATGGTCCTGCCC
PSV-VP2 F	CGCGAATTCAGCACCTGATAAGGAAGAAGAAGG
PSV-VP2 R	GACTGGTACCTTGTCTTGTTACAGACGATCTCAAGCC
PSV-VP3 F	GACAAGCTTGGTTTTCCAGTTAGACAAGTACCAG
PSV-VP3 R	GACTGGTACCTTGATAATTTGCAAAATAGGCAGTATCAGTGG
PSV-VP1 F	GACAAGCTTGGAGATGTAAAGGATGAGGTTCAGG
PSV-VP1 R	GACTGGTACCCAACTGCTCCGCTGAGTAAAAC
PSV-2A F	GACAAGCTTGGTCCCTATGAGATATGCCAGAC
PSV-2A R	GATGAATTCCTGGACCCAGTCATGCATTCC
PSV-2B F	GACAAGCTTGGACTCGGTCAGGTGTTTGG
PSV-2B R	TCCTCTAGATTACTGTTTATGTGGTTCTCCAGTTATCTTG
PSV-2C F	CGCGAATTCAGGACCCTCTGAGTGGCTTAAG
PSV-2C R	GACTGGTACCTTGAAAGATTGCATCAACCACATTCAG
PSV-3A F	CGCGAATTCAGGTCCAGTACAAGTGCCTGAG
PSV-3A R	GACTGGTACCTTGTTTTGAGGAGAACAACCTAACCATC
PSV-3B F	CGCGAATTCAGGAGCATACACTGGTGCACCTAAACCAGAGACTAGGAAACCTGTAC
PSV-3B R	GACTGGTACCCTGCACCACTGCTTTTCTGAGTACAGGTTTCCTAGTCTCTGG
PSV-3C F	CGCGAATTCAGGTCCTGACATGGAATTTGCC
PSV-3C R	GACTGGTACCTTGTTTGTTAACAAAATAATCTCTCTTCAGTAGGG
PSV-3D F	CGCGAATTCAGGATTGATAACAGAAAAATACACACCATC
PSV-3D R	ATCCTCTAGACTAAAACATATCTAACCAAGACCTACGC
2A-H58A F	GACAACAGCAGTTTTCCTCTGGAGAGCTTGGG
2A-H58A R	CCAGAGGAAAACTGCTGTTGTCTGACTCCAATTGTTAGCAG
2A-H125A F	TAAACTCTGCTGCATTCCCATGGAAACAGTACTCAGG
2A-H125A R	CCATGGGAATGCAGCAGAGTTTACAACTACTATATAATCACC
2A-C164A F	ATGGCTTCGCAGGAGCCGGTTTGATATCAAGG
2A-C164A R	CAAACCGGCTCCTGCGAAGCCATTATCAGCATCTCCAGC
2A-H48A F	GTACCCTACGCAGCTGCTAACAATTGGAGTCAGAC
2A-H48A R	GTTAGCAGCTGCGTAGGGTACCAATAATAGGTCTTCTCCC
2A-D91A F	CAAGGGCATTGACATTTCTTAAAATTGCATATGCTACACC
2A-D91A R	TAAGAAATGTCAATGCCCTTGTTGAATCTGTCCACATGTCTAC

### The TUNEL assay

The apoptotic cells were analyzed by the TUNEL Detection Kit (Beyotime). Briefly, ST or HEKHEK293A cells were cultured in 24-well plates. After PSV infected or pCAGGS-Flag-2A transfected, the cells were fixed with 4% paraformaldehyde and permeabilized using 0.2% Triton X-100 in 0.1% sodium citrate. Then, the cells were overlaid with TUNEL reaction mixture according to the manufacturer’ manual. Next, the cells were stained using VP1-specific mAb and Alexa Fluor 549-goat anti-mouse antibody. Finally, the cells were stained with DAPI and detected by the confocal microscope (Zeiss).

### Flow cytometric analysis

The cell apoptosis was determined using an FITC Annexin V Apoptosis Detection Kit (Solarbio) following the manufacturer’ instructions. Briefly, the cells were collected and washed twice with cold PBS by centrifugation at 300 × g for 5 min, and then stained with Annexin V and propidium iodide (PI). The percentage of apoptotic cells was examined by flow cytometry. At least 10^5^ events were recorded for each data.

### Analyses of the mitochondrial membrane potential

To analyze the mitochondrial membrane potential by the MitoProbe Assay Kit (Solarbio), HEK293A cells were transfected with pCAGGS-Flag-2A using different doses for 24 h. According to the manufacturer’ manual, the cells were incubated with JC-1 dye (2 nM) at 37°C for 30 min, and then cells were examined by flow cytometry using emission at wavelengths of 529 nm (green) and 590 nm (red). At least 10^4^ events were recorded for each data.

### Western blot analyses

The cells were seeded into six-well plates and collected with lysis buffer (Beyotime) containing protease and phosphatase inhibitors. The samples were then separated on a SDS–PAGE gel and transferred to PVDF membranes. The membranes were blocked with 5% skim milk in the Tris-buffered saline (TBS) containing Tween 20 for 2 h and then incubated with the primary antibodies at 4°C overnight. After three times washing with TBST, the membranes were incubated by HRP-conjugated secondary antibodies, and thereafter target proteins were visualized using an ECL system (Tanon). The gray density was performed using the ImageJ software.

### Immunofluorescence assay

The cells were mock-infected or infected with PSV. Subsequently, the cells were fixed with 4% paraformaldehyde and permeabilized with 1% Triton X-100 in PBS. Then, the cells were blocked with 1% BSA for 30 min. After washing three times in PBS, the cells were labeled with mouse anti-VP1 mAb at 4°C overnight. After another washing with PBS, the cells were incubated with Alexa Fluor 488-goat anti-mouse lgG for 1 h at RT, followed by staining with DAPI. Fluorescence images were captured using the confocal microscope (Zeiss).

### Cytosolic and mitochondrial fractionation

To isolate the cytosolic and mitochondrial fractions, the Mitochondria/Cytosol Fractionation Kit (Beyotime) was used. According to its manufacturer’s manual. HEK293A cells were transfected with pCAGGS-Flag-2A using different doses. After 24 h, the cells were harvested though centrifugation at 600 × g for 10 min at 4°C. After washing in the cold PBS, the centrifuged cells were resuspended in lysis buffer containing protease inhibitors. Subsequently, the cell suspensions were homogenized with the glass homogenizers. After 10-30 times of homogenization, the complete homogenates were centrifuged at 600 × g for 10 min at 4°C, and then transferred the supernatants to new tubes for another centrifugation at 12,000 × g for 10 min at 4°C. The supernatants were harvested as the cytosolic fraction, while the pellet was collected as mitochondrial fraction using mitochondrial lysis buffer.

### Statistical analysis

Data were shown as the mean ± standard deviation (SD). Results were performed by one-way ANOVA. Significance was analyzed using GraphPad Prism software. Differences were considered statistically significant when the p-value (*) was less than 0.05 and highly significant when the p-value (**) was less than 0.01; ns indicated not significant.

## Results

### PSV infection induced apoptosis in ST cells and HEK293A cells

**Figure 1:**
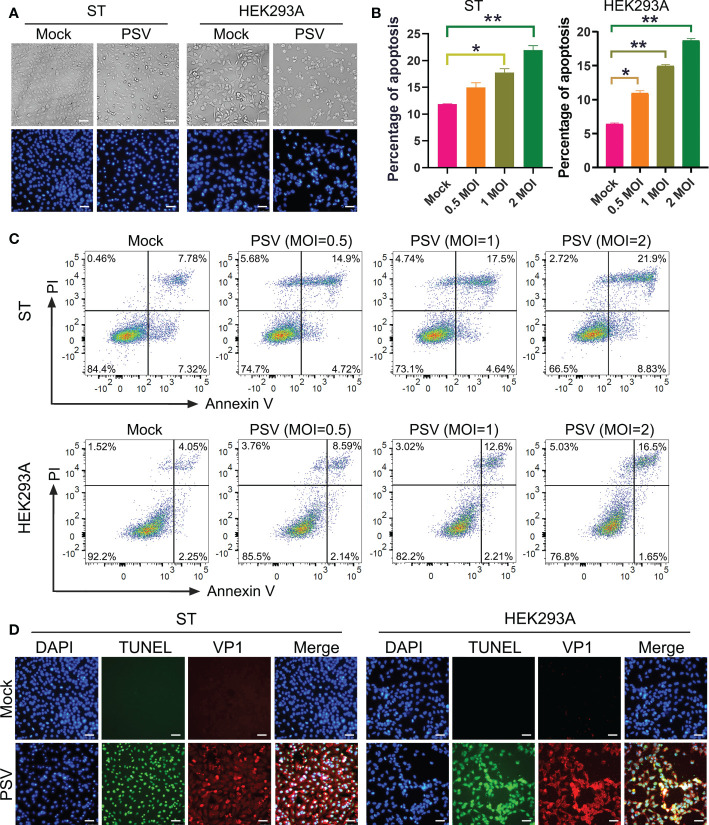
PSV infection induces apoptosis. **(A)** The cells infected with PSV (MOI=1) for 24 h were stained with DAPI, and then viewed under fluorescence microscope. **(B, C)** PSV-infected cells with different MOI (0.5, 1, and 2) were collected and subjected to dual Annexin V and propidium iodide (PI) labelling, and then detected by flow cytometry. The graph represented the percentage of Annexin V positive quadrants (n=3). *P < 0.05, **P < 0.01. **(D)** Mock and PSV-infected cells (MOI=1) were fixed at 24 hpi, sequentially, labelled with TUNEL (green), anti-PSV-VP1 antibody (red), and DAPI. The TUNEL labelling and VP1 protein staining cells were observed using fluorescence microscope. Scale bar: 100 μm.

To investigate whether PSV could trigger cell apoptosis, PSV-infected cells were observed by optical microscopy after staining with DAPI. The CPEs were visible in PSV-infected ST and HEK293A cells at 24 hpi and there were a marked cell shrinkage, nuclear diameter reduction and chromatin condensation in most of the infected cells (**Figure 1A**). Then, the intracellular fragmented DNA in PSV-infected cells were detected by the TUNEL assay. The results showed that the TUNEL positive signals were only exanimated in PSV-infected cells (MOI = 1, 24 hpi) (**Figure 1D**). To detect the precise apoptotic rate, the ST and HEK293A cells were infected with rising doses of PSV and stained with Annexin V/PI. The percentages of the viable, apoptotic, and necrotic cells were quantitatively determined by flow cytometry. PSV infection produced readily apparent apoptosis (Annexin V positive) at 24 hpi, compared with the mock-infected cells. The percentage of apoptotic cells increased with the dose of viral-infection and reached 30.73% and 18.15% in the ST and HEK293A cells, respectively (**Figures 1B, C**). These data demonstrated that PSV infection could induce cell apoptosis.

### Caspases were involved in PSV-induced apoptosis

**Figure 2:**
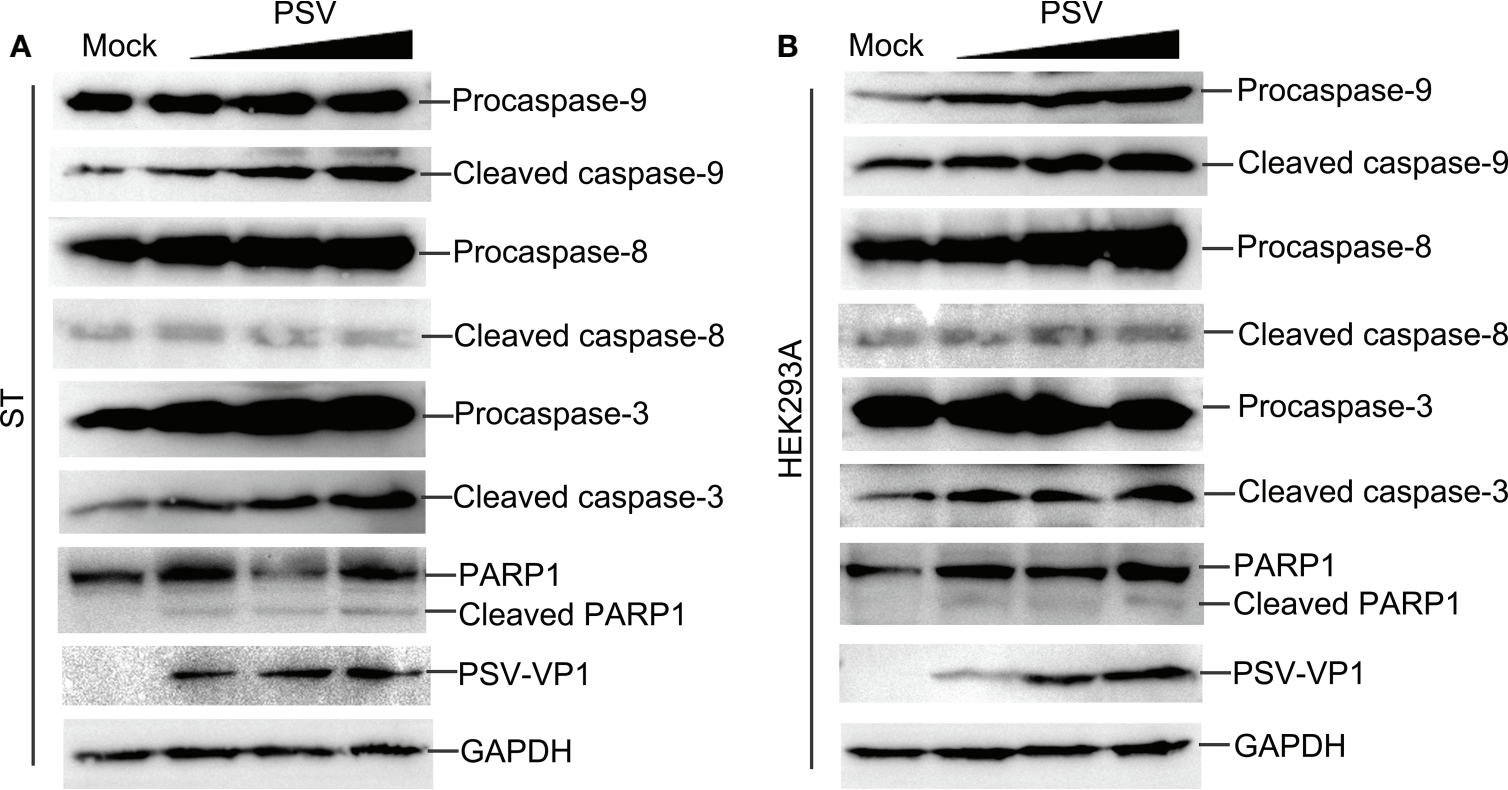
PSV infection induces caspase activation and PARP1 cleavage. **(A, B)** PSV-infected cells with different MOI (0.5, 1, and 2) were collected at 24 hpi. The activation of caspase-8, -9, -3 and the cleavage of PARP1 were analyzed by Western blot. GAPDH was used as internal loading control.

**Figure 3:**
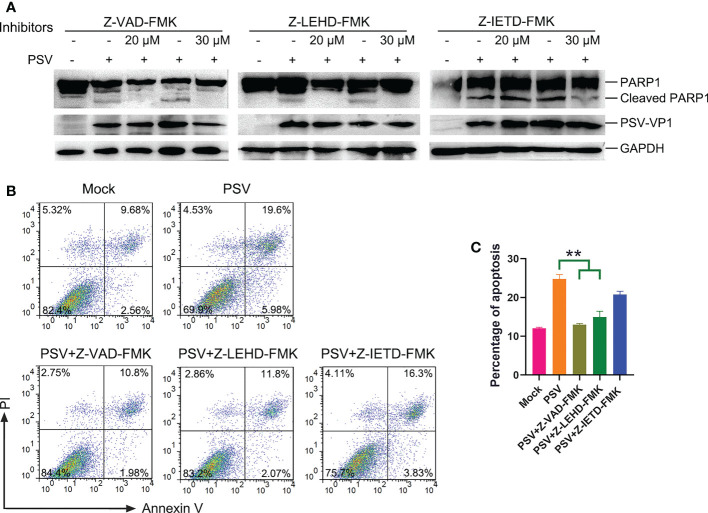
Caspase inhibitors affect PSV-induced apoptosis. **(A)** ST cells were pretreated with caspase inhibitors (Z-VAD-FMK, Z-LEHD-FMK, and Z-IEHD-FMK) (20 or 30 μM) and infected with PSV. After 24 h, the cellular lysates were detected by Western blot using antibodies against PSV-VP1 and PARP1. **(B, C)** ST cells were pretreated with caspase inhibitors at 30 μM, followed by PSV infection. Cells were harvested at 24 hpi, stained with Annexin V and PI, and then examined through flow cytometry. The graph represented the percentage of Annexin V positive quadrants (n = 3). **P < 0.01.

Caspase is a cysteine protease, which plays a basic role in cell apoptosis response to different stimuli (17, 18). The mitochondrial and death receptor mediated pathways are respectively induced by caspase-9 and caspase-8 (19). The contribution of caspases to PSV-induced apoptosis in ST and HEK293A cells were detected. As shown in **Figures 2A, B**, PSV infection promoted the cleavage of caspase-3 and caspase-9 at 24 hpi. In addition, the cleaved fragment of DNA repair enzyme PARP1, a substrate for caspase-3, increased with the dose of PSV infection. Furtherly, ST cells were pretreated with broad-spectrum caspase inhibitor (Z-VAD-FMK), specific inhibitor of caspase-8 (Z-IETD-FMK), or specific inhibitor of caspase-9 (Z-LEHD-FMK), and then infected with PSV for 24 h. The immunoblot results showed that pretreatment with Z-VAD-FMK and Z-LEHD-FMK significantly decreased PSV-induced cleavage of PARP1 (**Figure 3A**). In addition, the percentages of Annexin V positive cells were significantly reduced after pretreatment with Z-VAD-FMK or Z-LEHD-FMK, compared with PSV-infected cells alone (**Figures 3B, C**). These results suggest that caspase-3 and caspase-9 were activated by PSV infection.

**Figure 4:**
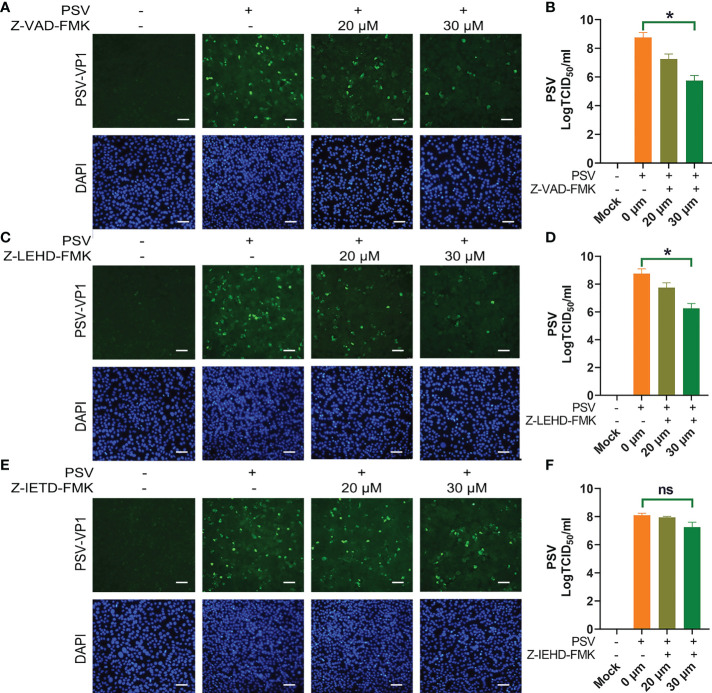
Caspase inhibitors affect PSV infection. **(A)** ST cells were treated with Z-VAD-FMK and infected with PSV. After 24 h, cells were fixed and incubated with anti-PSV-VP1 antibody and DAPI, followed by examined using fluorescence microscope. Scale bar: 100 μm. **(B)** After 36 h, the viral titers were determined by the Spearman-Kärber method, and three independent experiments were expressed as the mean ± SD. *P < 0.05. **(C-F)** The PSV replication in the presence of Z-LEHD-FMK and Z-IEHD-FMK. *P < 0.05; ns, no significant difference.

To explore the linkage between apoptosis and PSV replication. ST cells were pretreated with apoptosis inhibitors, followed by PSV infected at MOI = 1. The immunofluorescence and VP1 protein expression were detected at 24 hpi, and the results indicated that Z-VAD-FMK and Z-LEHD-FMK inhibited the viral replication (**Figures 4A, C, E**). Moreover, the viral titers were determined by TCID_50_ at 48 hpi, and caspase-3 and caspase-9 inhibitors caused significant reduction in viral load (**Figures 4B, D, F**). These results reveal that PSV replication is decreased after pretreatment with caspase-3 and caspase-9 inhibitors.

### Identification of viral proteins inducing apoptosis

**Figure 5:**
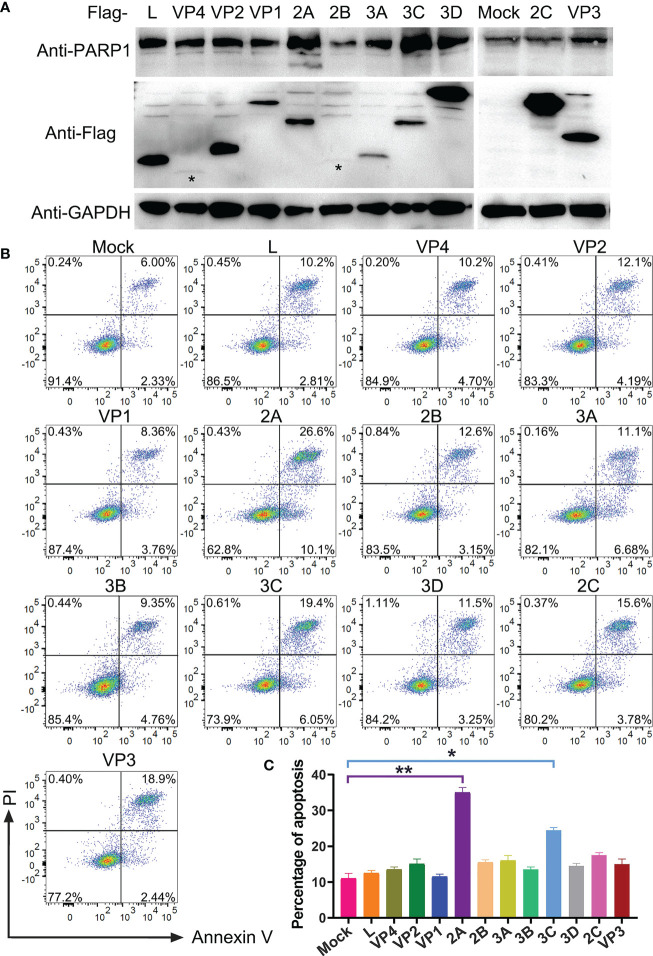
Identification of PSV-encoded proteins that induce apoptosis. **(A)** The expression plasmids of all PSV-encoded proteins were constructed and respectively transfected into HEK293A for 24 h. Then the cellular lysates were detected by Western blot with antibodies against Flag and PARP1 to detect the expression levels of viral proteins and the PARP1 cleavage. The asteroids indicate VP4, 2B protein bands. **(B, C)** The HEK293A cells were transfected with the expression plasmids of all PSV-encoded proteins, respectively. After 24 h, the cells were harvested and subjected to dual Annexin V and PI labelling, sequentially, detected by flow cytometry. The graph represented the percentage of Annexin V positive quadrants (n = 3). *P < 0.05, **P < 0.01.

After confirming that PSV infection resulted in apoptosis, the putative PSV-encoded protein(s) that may be responsible for the induction of apoptosis were tried to identify. The expression plasmids of all PSV-encoded proteins were constructed and transfected them into HEK293A. The expression of viral proteins were examined by Western blotting using anti-Flag antibody, and all genes were expressed as anticipated (**Figure 5A**). The cleaved PARP1 in 2A-transfected cells was detected by Western blotting (**Figure 5A**). Furthermore, the cell apoptosis induced by individual viral proteins was detected by flow cytometry. 2A and 3C produced a significant level of apoptosis, the percentage of Annexin V positive reached 36.7% and 25.4%, respectively (**Figures 5B, C**). The results indicated that 2A appeared to be one of the most potent inducer of apoptosis among the viral proteins.

### The 2A protein triggers the mitochondrial apoptotic pathway

**Figure 6:**
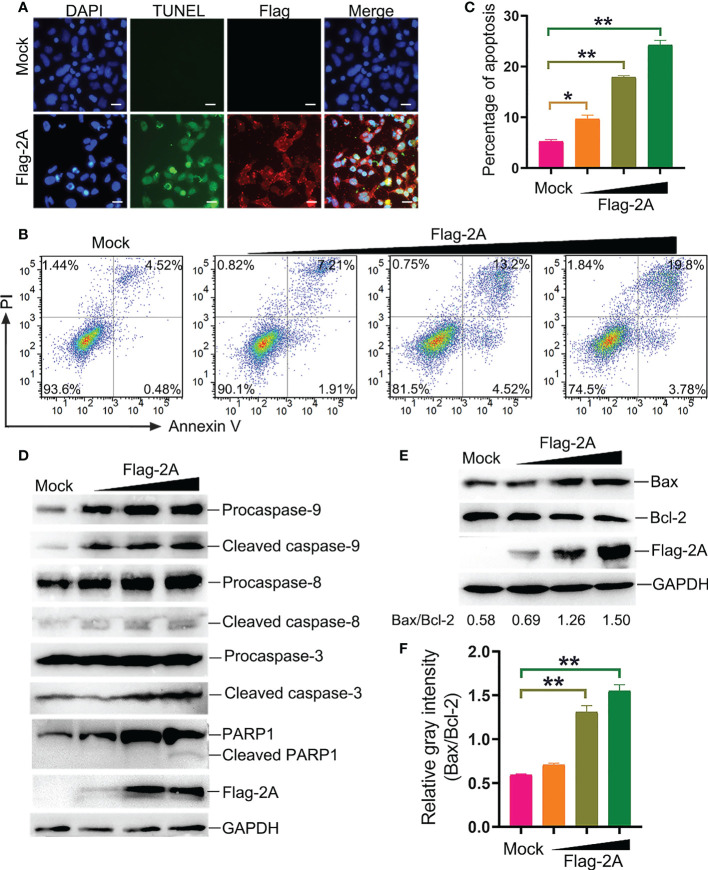
PSV 2A induces apoptosis. **(A)** The PSV 2A-transfected cells were fixed and incubated with TUNEL (green), anti-Flag antibody (red), and DAPI at 24 h, and then observed under fluorescence microscope. Scale bar: 100 μm. **(B, C)** The 2A-transfected cells were stained with Annexin V/PI, and detected by flow cytometry analysis. The graph represented the percentage of Annexin V positive quadrants (n = 3). *P < 0.05, **P < 0.01. **(D)** The activation of caspase-8, -9, -3 were analyzed *via* Western blot, after pCAGGS-Flag-2A transfected for 24 h. **(E, F)** The activation of Bax and Bcl-2 in 2A-transfected cells were detected at 24 h by Western blot. Ratios of the gray density of Bax and Bcl-2 were analyzed by Image J software. Densitometric data for Bax/Bcl-2 from three independent experiments are expressed as the mean ± SD. **P < 0.01.

To further assess the pathway of 2A-induced apoptosis, the TUNEL, Annexin V/PI, and immunoblotting assays were used to detect apoptosis in PSV-2A transfected cells. The results showed that TUNEL fluorescence intensity of the pCAGGS-Flag-2A transfected cells was enhanced, and Annexin V positive cells also significantly increased in the 2A-transfected cells (**Figures 6A-C**). In addition, the abundance of the cleaved fragments of caspase-3, caspase-9, and PARP1 detected by Western blotting was increased with the transfection dose in the 2A-transfected cells at 24 h (**Figure 6D**).

It is well known that Bax is one of the major pro-apoptotic factors which increases the mitochondrial outer membrane permeability, while Bcl-2 is an anti-apoptotic member promoting cell survival (20). The balance between Bax and Bcl-2 is necessary for the integrity of the mitochondrial membrane permeability. Bax and Bcl-2 were detected after pCAGGS-Flag-2A transfection. The results showed that 2A activated Bax and suppressed the Bcl-2 expression at 24 h (**Figure 6E**). The gray density of Bax/Bcl-2 was analyzed and the results showed that the ratio of Bax/Bcl-2 was increased after pCAGGS-Flag-2A transfection (**Figure 6F**).

**Figure 7:**
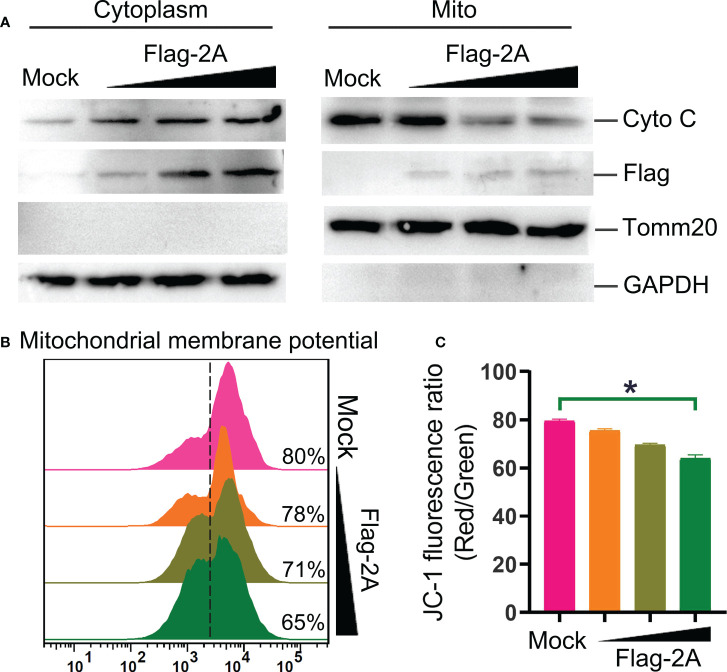
PSV 2A induces cytochrome C release and loss of mitochondrial membrane potential. **(A)** The cytosolic and mitochondrial fractions of PSV 2A-transfected HEK293A cells were isolated, and then the cytochrome C (cyto C) in cytosolic and mitochondrial fractions were analyzed by Western blot. **(B, C)** For detecting the mitochondrial membrane potential, the HEK293A cells were transfected with pCAGGS-Flag-2A for 24 h, the cells were labelled with JC-1 and detected through flow cytometry. The graph represents the JC-1 fluorescence ratio (Red/Green). *P < 0.05.

Furthermore, release of cyto C into the cytosol is critical for intrinsic apoptotic stimulation (21). We also observed that cyto C was decreased in the mitochondrial fraction and concomitantly increased dramatically in the cytosol (**Figure 7A**). Meanwhile, significant loss of the mitochondrial membrane potential in the 2A-transfected cells was observed (**Figures 7B, C**). These were further confirmed that 2A triggered the mitochondrial apoptotic pathway.

### The crucial residues of 2A protein for the induction of apoptosis

**Figure 8:**
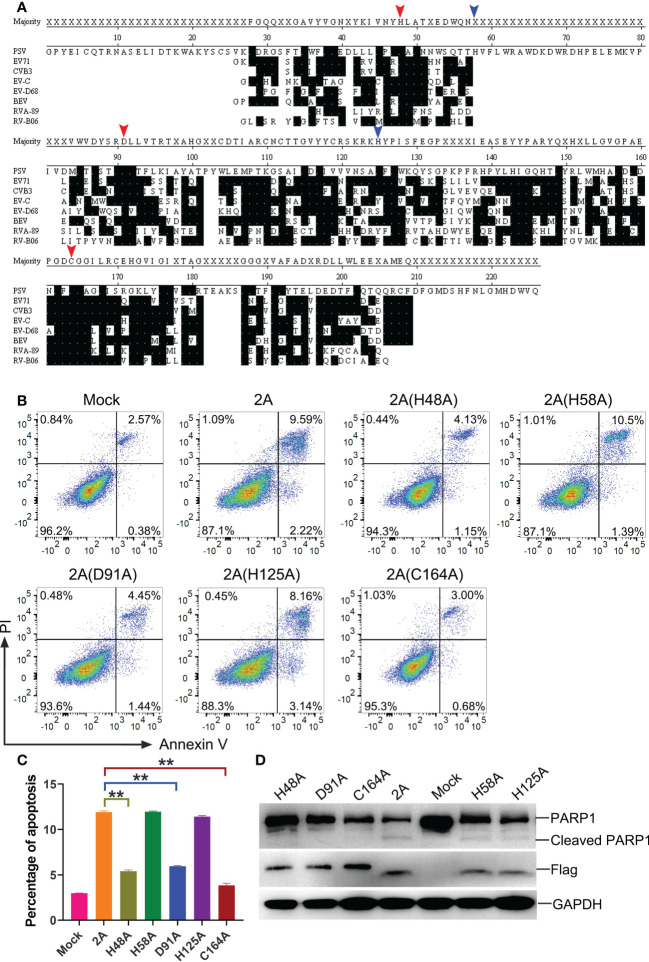
The crucial residues of PSV 2A for inducing the apoptosis. **(A)** The amino acid alignments of 2A in representative members of picornaviruses were listed. Identical residues are indicated by dots on black background. The residues marked with red arrow represent the amino acids that form the catalytic triad of poliovirus 2A. The residues marked with blue arrow are other amino acids selected as control. Residues are numbered according to the amino acid sequence of EV71 MH685713, CVB3 MF678329, EV-C NP_740477.1, EV-D68 KX351830, BEV NC_001859, RVA-89 NC_001617, and RV-B06 JX193795. **(B, C)** The HEK293A cells were transfected with pCAGGS-Flag-2A and its mutants: pCAGGS-Flag-2A(H48A), pCAGGS-Flag-2A(H58A), pCAGGS-Flag-2A(D91A), pCAGGS-Flag-2A(H125A), and pCAGGS-Flag-2A(C164A), after 24 h, the cells were collected and subjected to dual Annexin V and PI labelling, sequentially, detected by flow cytometry. The graph represented the percentage of Annexin V positive quadrants (n = 3). **P < 0.01. **(D)** HEK293A cells were transfected with pCAGGS-Flag-2A and its mutants for 24 h. Then the cellular lysates were analyzed through western blot with antibodies against Flag and PARP1.

To further investigate the key residues of the PSV 2A protein, the amino acid sequences of 2A in representative members of picornaviruses were aligned *via* the software program DNASTAR (**Figure 8A**). Based on the comparisons, a catalytic triad comprising H48, D91, and C164 amino acids was identified in the PSV 2A (marked with red arrow in **Figure 8A**). These were also conserved residues. In addition, we selected two amino acids, H58 and H125, as controls (marked with blue arrow in **Figure 8A**). Among the 2A mutants, 2A (H58A) and 2A (H125A) appeared to induce apoptosis and PARP1 cleavage. The H48, D91, and C164 mutated proteins suppressed the 2A-induced Annexin V positive cells and PARP1 cleavage (**Figures 8B-D**). These results suggested that the conserved residues H48, D91, and C164 in PSV 2A were crucial for the induction of apoptosis.

## Discussion

Apoptosis is a tightly regulated form of programmed cell death while this process occurs in response to diverse stimuli and conditions, such as viral infections (17). Viruses develop various mechanisms to induce apoptosis for facilitating promoting the viral replication and release, which are crucial biological activities in viral invasion and pathogenesis (17). Caspase cleavage exploited by viruses plays additional function to promote nuclear translocation of viral components, activate the host transcription regulators encoded by viruses, and facilitate the maturation of virion (22). Human Astrovirus-8 activated the initiator caspases-4, -8, and downstream executioner caspases-3, -7 for facilitating viral release (23). Picornaviruses can modulate host apoptosis, thus successfully proliferating (8). There are few reports on PSV infection and apoptosis. In our study, we found that infection of PSV triggered significant cell apoptosis, where chromatin condensation, nuclear fragmentation, phosphatidylserine (PS) eversion, and PARP1 degradation occurred. Studies have reported that viruses employed intrinsic, extrinsic, or both pathways for inducing apoptosis (24–26). In *Picornaviridae* family members, Senecavirus A (SVA) impacts the extracellular and intracellular pathways and then regulated the cell apoptosis (27). EV71 infection was reported to stimulate apoptosis cascade through the intrinsic apoptotic pathway in many cell lines (28, 29). The PV could induce mitochondrial Cytochrome C release and initiate cell apoptosis, thereby promoting virus replication (30). We first revealed that the PSV-induced apoptosis was mainly through mitochondrial apoptotic pathway, which depended on the activation of caspase-3 and caspase-9. In addition, the corresponding caspase inhibitors suppressed PSV replication indicating that apoptosis was required in the PSV lifecycle. It has been reported that PSV could infect the intestinal epithelial cells. Therefore, it can be speculated that PSV can induce the apoptosis in intestinal epithelial cells, which may be related to the symptom of acute diarrhea caused by this virus in swine.

The genome of picornaviruses contains a single large open reading frame that is translated into a polyprotein. To generate functional viral proteins, the polyprotein is cleaved by 2A and 3C proteases (31, 32). As reported previously, the proteases in *Picornaviridae* family members are not only essential for cleavage of viral polyprotein but also target cellular factors to induce apoptosis (33, 34). In picornaviruses, PV was capable of evoking the apoptotic reaction, with its 2A and 3C proteins being implicated in the apoptosis-inducing function (16, 35, 36). Depending on the protease activity, SVA 3C protease played a potential role in SVA-induced apoptosis and cleavage of NF-κB and PARP1 (27). In addition, EV71 3C and 2A proteins are both responsible for cleaving host cell proteins to trigger apoptosis (12). Recently, PSV 3C was reported to induce cell apoptosis but the specific mechanism was unclear (5). Through our investigation, the 2A protein was the most efficient apoptosis inducer among all PSV-encoded proteins, promoting the caspases and PARP1 cleavage, the pro-apoptotic factor expression, and mitochondrial abnormal structure. The interaction between 2A and the key apoptosis regulating proteins (such as Bax, Bcl-2, Bcl-xL, and MAVS) in the mitochondrial apoptotic pathway, and the mechanism of 2A-induced apoptosis needs to be further elucidated, which will contribute to understanding PSV pathogenesis and designing antiviral therapeutics.

As reported, several picornaviruses 2A protein were structurally similar to trypsin-like serine protease (12). Although the nature of picornaviruses 2A protease was unknown, researchers identified that 2A were similar in sequence to a class of small cellular serine proteases. Moreover, it was proved that the 2A catalytic triad was composed of His, Asp, and an active site nucleophile Cys (37). In the alignment of 2A amino acid sequences from several picornaviruses (**Figure 8A**), there were only two amino acids (H48 and C164) identified in the PSV 2A, which might be the composition of a putative triad. In addition, the amino acid D85 was another residue responsible for the catalytic triad as reported in the *Enterovirus* family (38). Accordingly, we speculated that the amino acid D91 in PSV 2A contributed to induce apoptosis, which was at the similar position with the amino acid D85 in the Enterovirus sequence. From our research, all of these three amino acids (H48, D91, and C164) were conserved, related to the cleavage activity, and essential for the 2A-induced apoptosis. Therefore, the PSV 2A-induced apoptosis depends on its protease activity.

In summary, we demonstrated that PSV infection triggered the intrinsic apoptosis pathway, thereby promoting viral production. Among the proteins encoded by PSV, 2A is an important factor in inducing the mitochondrial apoptotic pathway, and the conserved amino acids H48, D91, and C164 of 2A are essential for 2A-induced apoptosis. This study provides insights into the pathogenic mechanism of PSV and can aid in developing measures to prevent PSV infection.

## Data availability statement

The original contributions presented in the study are included in the article/supplementary material. Further inquiries can be directed to the corresponding author.

## Author contributions

CM and YW designed, performed, and analyzed the experiments and data. SP and KS developed reagents and helped with experiments. ZC planned experiments and secured funding. CM wrote the manuscript. All authors contributed to the article and approved the submitted version.

## Funding

This work was supported by the Key Research and Development Program of Guangxi, China (2021AB21037), the Agricultural Science and Technology Independent Innovation Fund of Jiangsu Province [CX(21)2014], the 111 Project D18007 and the Priority Academic Program Development of Jiangsu Higher Education Institutions (PAPD).

## Acknowledgments

We thank all of members at Dr. Chen’s lab for their suggestions and excellent technical assistance.

## Conflict of interest

The authors declare that the research was conducted in the absence of any commercial or financial relationships that could be construed as a potential conflict of interest.

## Publisher’s note

All claims expressed in this article are solely those of the authors and do not necessarily represent those of their affiliated organizations, or those of the publisher, the editors and the reviewers. Any product that may be evaluated in this article, or claim that may be made by its manufacturer, is not guaranteed or endorsed by the publisher.
